# Sustained Antibody-Dependent NK Cell Functions in Mild COVID-19 Outpatients During Convalescence

**DOI:** 10.3389/fimmu.2022.796481

**Published:** 2022-02-07

**Authors:** Francisco Fuentes-Villalobos, Jose L. Garrido, Matías A. Medina, Nicole Zambrano, Natalia Ross, Felipe Bravo, Aracelly Gaete-Argel, Aarón Oyarzún-Arrau, Fatima Amanat, Ricardo Soto-Rifo, Fernando Valiente-Echeverría, Renato Ocampo, Christian Esveile, Leonila Ferreira, Johanna Cabrera, Vivianne Torres, Maria L. Rioseco, Raúl Riquelme, Sebastián Barría, Raymond Alvarez, Yazmín Pinos, Florian Krammer, Mario Calvo, Maria I. Barria, COVID-19 South Chile Group

**Affiliations:** ^1^ Department of Microbiology, Faculty of Biological Science, Universidad de Concepción, Concepción, Chile; ^2^ Ichor Biologics LLC, New York, NY, United States; ^3^ Facultad de Medicina y Ciencia, Universidad San Sebastián, Puerto Montt, Chile; ^4^ Laboratory of Molecular and Cellular Virology, Virology Program, Institute of Biomedical Sciences, Faculty of Medicine, Universidad de Chile, Santiago, Chile; ^5^ Department of Microbiology, Icahn School of Medicine at Mount Sinai, New York, NY, United States; ^6^ Hospital Regional de Talca, Talca, Chile; ^7^ Hospital Clínico Herminda Martin, Chillán, Chile; ^8^ Hospital Clínico Regional Dr. Guillermo Grant Benavente, Concepción, Chile; ^9^ Hospital Dr. Hernán Henríquez Aravena, Temuco, Chile; ^10^ Institute of Medicine, Faculty of Medicine, Universidad Austral de Chile, Valdivia, Chile; ^11^ Hospital Puerto Montt Dr. Eduardo Schütz Schroeder, Puerto Montt, Chile; ^12^ Division of Infectious Diseases, Department of Medicine, Immunology Institute, Icahn School of Medicine at Mount Sinai, New York, NY, United States; ^13^ Hospital Base San José, Osorno, Chile

**Keywords:** Fc-effector functions, NK cells activity, spike (S) glycoprotein, SARS-CoV-2, COVID-19, outpatients

## Abstract

The coronavirus disease 2019 (COVID19) pandemic has left researchers scrambling to identify the humoral immune correlates of protection from COVID-19. To date, the antibody mediated correlates of virus neutralization have been extensively studied. However, the extent that non-neutralizing functions contribute to anti-viral responses are ill defined. In this study, we profiled the anti-spike antibody subtype/subclass responses, along with neutralization and antibody-dependent natural killer cell functions in 83 blood samples collected between 4 and 201 days post-symptoms onset from a cohort of COVID-19 outpatients. We observed heterogeneous humoral responses against the acute respiratory syndrome coronavirus 2 (SARS-CoV-2) spike protein. Overall, anti-spike profiles were characterized by a rapid rise of IgA and sustained IgG titers. In addition, strong antibody-mediated natural killer effector responses correlated with milder disease and being female. While higher neutralization profiles were observed in males along with increased severity. These results give an insight into the underlying function of antibodies beyond neutralization and suggest that antibody-mediated natural killer cell activity is a key function of the humoral response against the SARS-CoV-2 spike protein.

## Introduction

Severe acute respiratory syndrome coronavirus (SARS-CoV-2) infection results in a majority of symptomatic individuals presenting with mild disease (1–4). The individuals most frequently requiring hospitalization were those with underlying co-morbidities such as hypertension and diabetes (1, 3, 5). Interestingly, previous studies have reported that the severity of infection is associated with higher antibody (Ab) titers and neutralizing activity compared to individuals in whom the disease is mild or moderate (6–10). Despite early attention to hospitalized individuals (1, 5, 11), the still-growing population of outpatients with moderate to more severe symptomatology represents a critical group to study. Previously, they were reported to present a waning of spike-specific Ab titers during the first months after infection (12–15). Fortunately, while the quest for immune correlates of protection against SARS-CoV-2 infection continues (16) accumulating evidence is beginning to identify immune hallmarks related to the severity of symptoms reported by patients, and would contribute to knowing deeper aspects of the natural immune response expected to be replicated by prophylactic and therapeutic approaches (8, 17–22).

The use of Ab titers as the gold standard for measuring the acquired protection after vaccination or infection is common practice (23–25). However, the ability of Abs to control pathogen infection or protect against reinfection is not solely dependent on their titers, but rather on their capacity to induce neutralizing and non-neutralizing functions (26–30). Several studies have now explored the Ab correlates of SARS CoV-2 neutralization (8, 21, 31–35), in particular, Immunoglobulin (Ig) G class has received more attention, due to its well-known participation in neutralization induced by viral infections (36). Yet, the contribution of non-neutralizing Ab responses remains ill defined.

Beyond neutralizing functions, Abs can trigger non-neutralizing antigen-specific innate immune functions *via* interactions with Fc receptors found on the surface of innate immune cells, playing an essential role in connecting adaptive to innate immunity. Indeed, Abs mediating these functions have been recently discovered to play an important role against Ebola virus (29, 37), HIV (38, 39), and influenza virus (40, 41) infections. These Ab-dependent cellular functions include among others phagocytosis of infected cells and natural killer (NK) cell effector functions. After their activation, NK cells can control infection through both, the release of lytic granules containing perforin and granzymes; and the release of proinflammatory cytokines like IFN-γ and TNF-α (42, 43). Those functions can be induced after the binding of different Fc receptors to Fc domains of Abs engaged to viral proteins presented in infected cells (44, 45). Notably, Abs commonly have the potential to elicit several effector functions and diverse determinants have been found to modulate their activation, such as Ab isotype, subclass, glycosylation pattern and specificity for viral antigens on the Ab side, and, differential Fc receptors expression and polymorphisms found in effector cells, among other factors (46). In COVID-19, both Fc effector functions previously mentioned have been correlated with immunity in SARS-CoV-2 vaccine studies using animal models (47, 48). Moreover, Ab-dependent Fc effector activity appears to be compromised in deceased versus recovered individuals (19). A recent paper has also described the contribution of neutralizing and Fc functions in seroconverted individuals after asymptomatic/mild infection (27). These studies highlight that the breadth of Ab functionality, rather than Ab titers, is more correlated with immunity (45, 49). Therefore, it is important to examine the contribution of Ab responses beyond neutralization and define if non-neutralizing Ab responses are associated with mild or more severe infections. It will further be important to define whether or not natural infection in outpatients induces sustained Ab response that yields protective and long-lived immunity. Considering that these patients are the majority of cases reported during this pandemic, along with a growing portion of convalescent population mainly from non-hospitalized cases, it is essential to investigate their immune correlates of protection post infection.

In the present study, we quantified IgA, IgM, and IgG Abs against SARS-CoV-2 spike protein and its receptor binding domain (RBD), and measured the Ab neutralization and Ab-dependent NK effector functions against SARS-CoV-2 spike in a cohort of 70 outpatient individuals. Correlations and principal component analyses allowed us to determine the contribution of the studied parameters and their association with symptoms severity, days post-symptoms onset and demographic variables such as sex. Interestingly, while NK effector functions were associated with individuals that developed a mild disease, specifically females; neutralization was most represented in the group of individuals who suffered more severe symptoms, specifically males. In summary, our data propose a humoral signature associated with milder disease in coronavirus disease 2019 (COVID-19) outpatients.

## Materials and Methods

### Sample Cohort

Healthy controls were obtained from a cohort of subjects, whose samples were collected prior to the COVID-19 pandemic (50). Samples were processed as previously described (51). Serum from each donor was obtained from serum collection tubes (BD Vacutainer) and stored at -80°C.

Clinical symptoms were classified in five categories: systemic (fever, headache, myalgia, chills), upper respiratory (odynophagia), lower respiratory (cough, dyspnoea), neurological (anosmia, dysgeusia), and gastrointestinal (diarrhea), to determine a clinical severity score, we analyzed, the number of categories affecting each subject generating a score from 1 to 5 for symptomatic cases.

### SARS-CoV-2 Spike and RBD Recombinant Protein Expression and Purification

The previously characterized SARS-CoV-2 spike and RBD were produced as previously described (52, 53). Briefly, codon-optimized coding sequences based on SARS-CoV-2 isolate (GenBank: MN908947.3) for 6xHistidine-tagged soluble RBD and spike (at trimeric pre-fusion conformation), were cloned into pCAGGS backbones, transfected into HEK293 T cells, and subsequently purified from supernatants throughout Ni-NTA Agarose (Qiagen). Recombinant proteins were then concentrated using Amicon centrifugal units (EMD Millipore) and re-suspended in 0.067M PO_4_ pH 7.4 phosphate-buffered saline (PBS). Sodium dodecyl-sulfate polyacrylamide gel electrophoresis (SDS-PAGE) and Coomassie staining were used to confirm protein stability.

### Spike- and RBD- Enzyme-Linked Immunosorbent Assays (ELISAs)

Based on protocols from a previous study (53), 96-well plates were coated with 2 µg/ml solution of soluble trimeric spike ectodomain or RBD purified proteins at a volume of 50 µl per well overnight. The plates were then washed and blocked with 3% non-fat milk prepared in PBS with 0.1% Tween 20 (PBST). Serial dilutions of previously heated serum samples were prepared in 1% non-fat milk prepared in PBST and incubated in the washed coated plates for 2 h at room temperature. The plates were washed again and incubated with a 1:3,000 dilution of goat anti-human IgG, IgA, IgM –horseradish peroxidase (HRP) conjugated antibodies (Thermo Fisher Scientific) for IgG1, IgG2, IgG3, or IgG4, followed by incubation with secondary anti-mouse HRP antibody (Jackson ImmunoResearch). All antibodies were prepared in 0.1% PBST and incubated for 1 hour. After a final wash, 100 µl of SIGMAFAST OPD (o-phenylenediamine dihydrochloride; Sigma–Aldrich) substrate solution was added to each well for 10 min, the reactions were then stopped with 3N hydrochloric acid. The optical density at 490 nm (OD490) was measured using a TECAN Infinite M Nano plate reader. Data were analyzed using Prism (GraphPad).

### Pseudovirus Neutralization Assay

Generation of the HIV-1-SΔ19 pseudotyped viral particles and HEK-ACE2 (human embryo kidney cells expressing angiotensin converting enzyme 2) cells were carried out as previously described (54). For neutralization assays, serum samples were initially diluted 1:40 in Dulbecco’s modified Eagle’s medium (DMEM) and serially diluted 1:3 up to a dilution of 1:87480. Simultaneously, a 96 white well-plate was loaded with 50 µL of HIV-1-SΔ19 pseudotyped viral particles containing approximately 4.5 ng of p24 equivalents diluted in DMEM. Subsequently, 50 µL of each serum dilution point was added to the particle-loaded wells in duplicate and mixed. As a positive control, 50 µL of DMEM were mixed with pseudotyped particles.

Plates were incubated for 1 hour at 37°C and then 100 µL of DMEM containing 1x10^4^ HEK-ACE2 cells was added to each well. As a negative control, HEK293T cells were added to pseudotype-containing wells. Firefly luciferase activity was measured 48 hours later, according to manufacturer’s instructions (E1980, PROMEGA) using a GLOMAX luminometer.

Estimation of the 50% neutralization titer (NT50) was obtained using a 4-parameter nonlinear regression curve fit measured as the percent of neutralization determined by the difference in average relative light units (RLU) between test samples and pseudotyped virus controls.

### Antibody-Mediated Degranulation and Activation of Human NK Cells

Human NK cells were enriched from fresh peripheral blood of healthy human volunteer donors’ buffy coats, obtained by Clínica Sanatorio Alemán blood bank at Concepción, Chile.

Briefly, a negative selection kit (Miltenyi) was used to isolate NK cells. 2 µg /ml of SARS-CoV-2 spike recombinant proteins were coated on ELISA High Bind Microplate (Corning) at 4°C overnight, and plates were blocked with 5% bovine serum albumin (BSA) prior to addition of the serum dilutions (1/100) in PBS for 2 hours at 37°C. Unbound antibodies were removed by washing wells 3X with PBS prior to the addition of NK cells. The NK cells were added at 5 x 10^5^ cells/well in the presence of brefeldin A (BioLegend), monensin (BioLegend), and anti-CD107a phycoerythrin (PE) (BioLegend) and incubated for 5 hours at 37°C. NK cells were surface stained with CD56 Alexa Fluor 647 (BioLegend), followed by intracellular staining with IFNγ Alexa Fluor 488 (BioLegend) and MIP1β Brilliant Violet 421 (BioLegend) using the Fix & Perm cell permeabilization kit (Invitrogen) used to detect the production of cytokine/chemokine. Cells were analyzed on a BD LSRFortessa X-20 flow cytometer and data was analyzed using FlowJo software (37).

### Statistics

Statistical and data analyses were performed using GraphPad Prism 8.4.3, R 4.0.4, and R Studio 1.4.1103. Graphs were generated in both Prism and R Studio, some of these were adjusted for better visualization using the software Adobe Illustrator (23.0.1).

**Figure 1:**
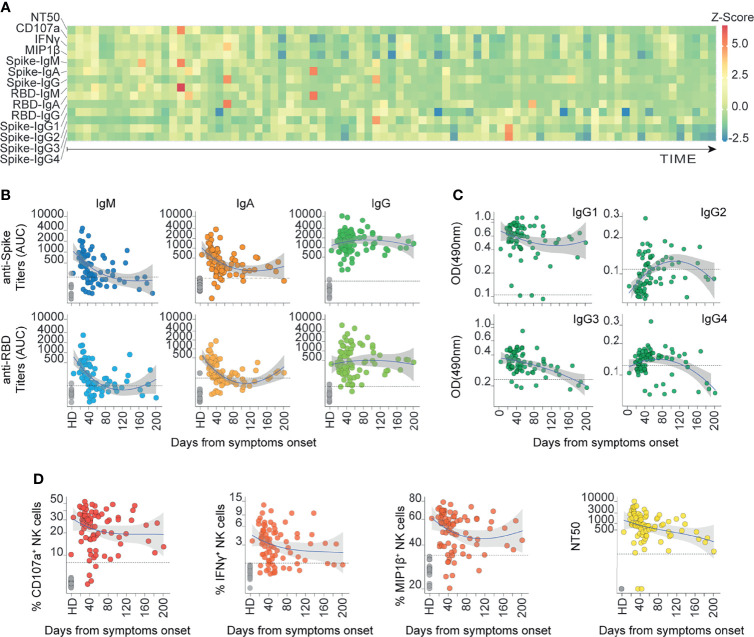
The humoral response mounted against SARS-CoV-2 Spike is heterogeneously represented up to 6 months after the onset of symptoms in COVID-19 outpatients. **(A)** Heatmap showing the humoral immune response for each patient ordered from the earliest to the latest collected sample (83 COVID-19 samples from 70 donors). The color scale represents the Z-scores calculated independently for each variable. **(B)** Spike and RBD-specific titers for IgM, IgA, and IgG antibodies measured as AUC (Area Under Curve) are displayed according to their respective sampling day from the onset of symptoms (83 COVID-19 samples from 70 donors and 14 controls). Loess estimation curve for COVID-19 samples is displayed in blue with gray-area indicating standard-error. Dotted line indicates the threshold of mean values from healthy donors plus two standard deviations. **(C)** Spike-specific IgG1, IgG2, IgG3, and IgG4 sub-class Abs measured as OD490 values are displayed according to their respective sampling day from the onset of symptoms (83 COVID-19 samples from 70 donors and the mean of controls). Loess estimation curve for COVID-19 samples is displayed in blue with gray-area indicating standard-error. Dotted line indicates the threshold of mean values from healthy donors. **(D)** Spike-specific functions of sera are displayed according to their respective sampling day from the onset of symptoms. Ab-dependent NK degranulation, measured as the frequency of CD107a^+^ NK cells; Ab-dependent NK activation, measured as the frequency of IFNγ^+^ and MIP1β^+^ NK cells; plus their neutralizing ability, measured as NT50 values (83 COVID-19 samples from 70 donors and 14 controls). Loess estimation curve for COVID-19 samples is displayed in blue with gray-area indicating standard-error. Dotted line indicates the threshold of mean values from healthy donors plus two standard deviations. Dotted line in NT50 indicates the reciprocal of the initial sera dilution used in the assay.

**Figure 2:**
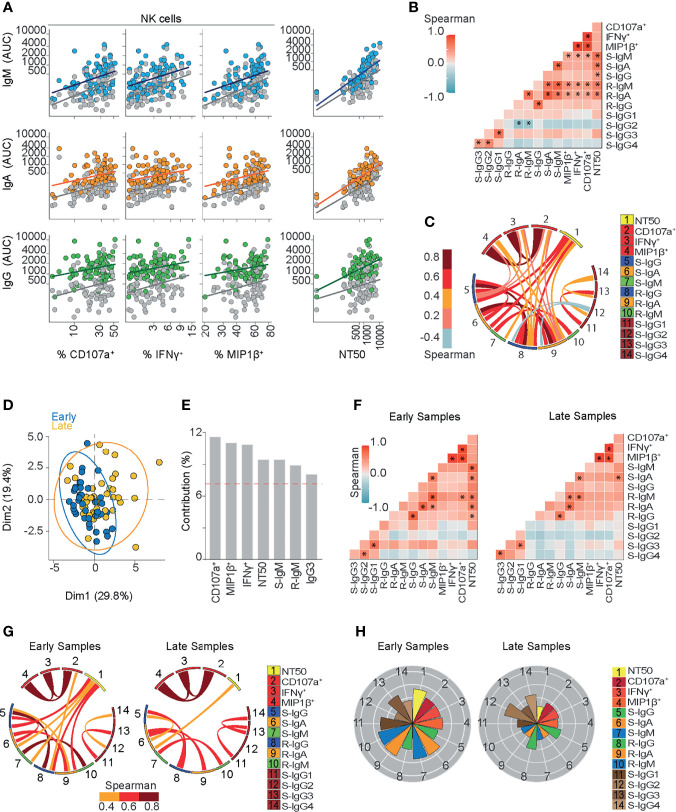
Ab-dependent NK effector functions are represented during the early phase and shown to be sustained six months after symptoms onset in COVID-19 outpatients. **(A)** A scatter plot showing linear regressions for Spike and RBD-specific titers for IgM, IgA, and IgG antibodies shown as AUC (Area Under Curve) values against their respective Ab-dependent functions: Ab-dependent NK degranulation, shown as the frequency of CD107a^+^ NK cells; Ab-dependent NK activation, shown as the frequency of IFNγ^+^ and MIP1β^+^ NK cells; plus neutralizing ability, shown as NT50 values (83 samples from 70 donors). Colored or gray circles display Spike or RBD-specific titers, respectively. **(B)** Correlation heatmap showing the Spearman’s r values between the complete set of variables analyzed for the entire cohort of COVID-19 outpatients (83 samples from 70 donors). The scale of blue-to-red color indicates a negative-to-positive correlation. Asterisks are shown for statistical significance of Holm-Bonferroni adjusted for multiple hypothesis testing (*p < 0.05). **(C)** Circos plot showing the significant Spearman correlations between the pairs of variables displayed with asterisks in **(B)**. The color of the links represents the magnitude of Spearman’s r values. **(D)** Biplot showing the principal component analysis (PCA) depicting the “early” and “late” convalescence samples distinguished based on their sampling from the onset of symptoms (Early ≤ 43 days; Late > 43 days) (83 samples from 70 donors). **(E)** Contribution of variables for both dimensions (1 and 2) in the PCA analysis. **(F)** Correlation heatmap showing the Spearman’s r values between the complete set of variables analyzed for early-sampled (42 samples from 42 donors) and late-sampled (41 samples from 31 donors) groups of outpatients. A scale of blue-to-red color indicates a negative-to-positive correlation. Asterisks are shown for statistical significance of Holm-Bonferroni adjusted for multiple hypothesis testing (*p < 0.05). **(G)** Circos plot showing the significant Spearman correlations between the pairs of variables displayed with an asterisk in **(F)** for early-sampled and late-sampled groups of outpatients. The color of the links represents the magnitude of Spearman’s r values. **(H)** Polar plots showing the differential composition of humoral immune responses in different groups. Each bar represents the mean z-scores of variables for early-sampled (42 samples from 42 donors) or late-sampled (41 samples from 31 donors) outpatients.

**Figure 3:**
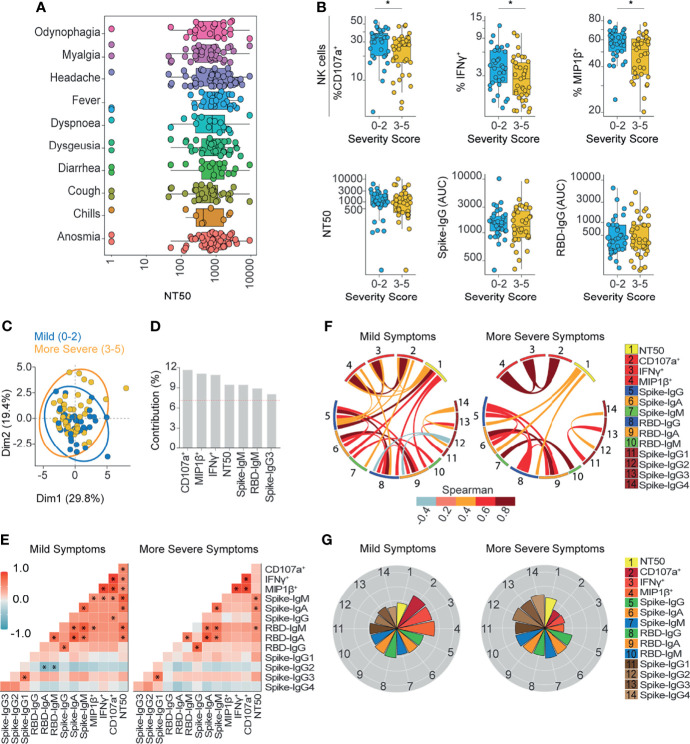
Ab-dependent NK effector functions are prominently enriched in subjects with mild symptoms compared to subjects with more severe symptoms outpatients. **(A)** Box plots showing the neutralization function activity grouped according to different symptoms reported by patients at the time of diagnosis (70 donors). **(B)** Box plots showing differentially enriched functions and Ab titers according to the severity score of symptoms. Statistical differences were evaluated between groups of outpatients classified as 0-2 (33 samples and 33 donors) or 3-5 scores (37 samples and 37 donors), using two-tailed unpaired nonparametric Mann-Whitney test (*p value < 0.05). **(C)** Biplot showing the principal component analysis (PCA) depicting the “mild” (39 samples and 33 donors) and “more severe” (44 samples and 37 donors) COVID-19 outpatients, distinguished based on their severity scores, between 0 and 2 (mild) or between 3 and 5 (more severe). **(D)** Contribution of variables for both dimensions (1 and 2) in the PCA analysis. **(E)** Correlation heatmap showing the Spearman’s r values between the complete set of variables analyzed for mild-scored (33 samples from 33 donors) and more severe-scored (37 samples from 37 donors) groups of outpatients. The scale of blue-to-red color indicates a negative-to-positive correlation. Asterisks are shown for statistical significance of Holm-Bonferroni adjusted for multiple hypothesis testing (*p < 0.05). **(F)** Circos plot showing significant Spearman correlations between the pairs of variables displayed with an asterisk in **(E)** for mild-scored and more severe-scored groups of outpatients. The color of the links represents the magnitude of Spearman’s r values. **(G)** Polar plots showing the differential composition of humoral immune responses at different groups. Each bar represents the mean z-scores of variables for mild-scored (33 samples from 33 donors) or more severe-scored (37 samples from 37 donors) outpatients.

Scatter plots in **Figures 1**–**3** and box plots in **Figure 3** were visualized with ggplot2 (v3.3.3) in R Studio. Statistical differences for boxplot in **Figure 3B** were calculated by Mann-Whitney test. Statistical significance was defined as * p < 0.05.

The heatmap in **Figure 1** was generated using the function “geom_tile” in R studio to represent the enrichment profile for each sample. Previously, all data were normalized to z-score where each variable was mean-centered and then divided by the standard deviation of the variable.

**Figure 4:**
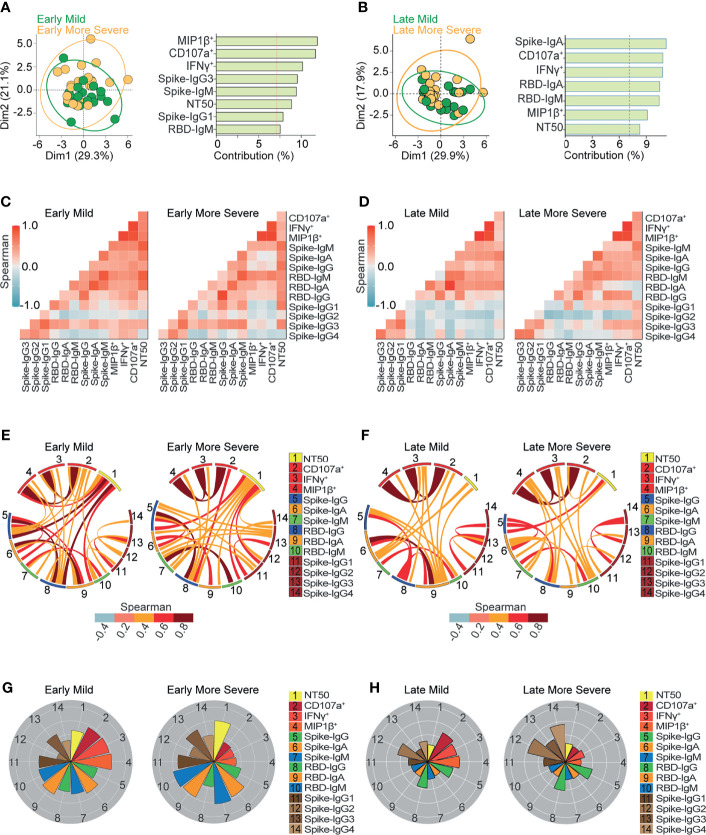
Ab-dependent NK effector functions are prominently enriched in mild compared to more severe outpatients up to 6 months from the onset of symptoms. **(A, B)** Biplot showing the principal component analysis (PCA) depicting the early-mild (21 samples from 21 donors) and early-more severe (21 samples from 21 donors) sub-groups of COVID-19 outpatients **(A)**; the late-mild (18 samples from 12 donors) and late-more severe (23 samples from 18 donors) sub-groups of COVID-19 outpatients **(B)**. The bar graph shows the contribution of variables for both dimensions (1 and 2) in the PCA analysis. **(C, D)** Correlation heatmap showing the Spearman’s r values between the complete set of variables analyzed for early-mild (21 samples from 21 donors) and early-more severe (21 samples from 21 donors) sub-groups of outpatients **(C)**; or for late-mild (18 samples from 12 donors) and late-more severe (23 samples from 18 donors) sub-groups of outpatients **(D)**. The scale of blue-to-red color indicates a negative-to-positive correlation. **(E, F)** Circos plot showing significant Spearman correlations without adjustment between the pairs of variables displayed in **(C)** or **(D)** for early-mild and early-more severe sub-groups of outpatients **(E)**; or for late-mild and late-more severe sub-groups of outpatients **(F)**. The color of the links represents the magnitude of Spearman’s r values. **(G, H)** Polar plots showing the differential composition of humoral immune responses at different sub-groups. Each bar represents the mean z-scores of variables for early-mild (21 samples from 21 donors) or more early-more severe outpatients (21 samples from 21 donors) **(G)**; and for late-mild (18 samples from 12 donors) or more late-more severe outpatients (23 samples from 18 donors) **(H)**.

**Figure 5:**
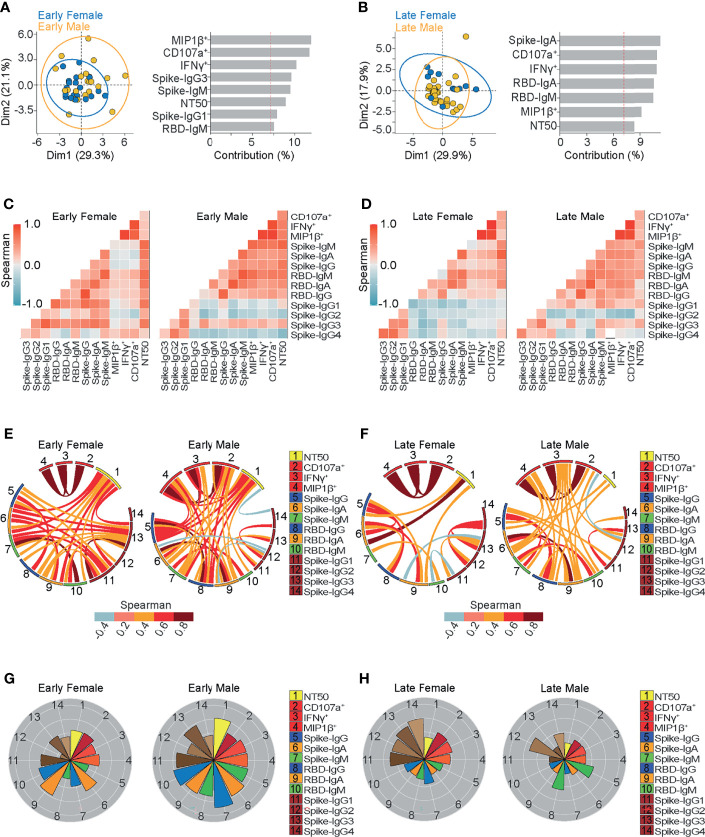
Ab-dependent NK effector functions but not neutralization, are better sustained in females compared to males up to 6 months from the onset of symptoms. **(A, B)** Biplot showing the principal component analysis (PCA) depicting the early-female (20 samples from 19 donors) and early-male (22 samples from 21 donors) sub-groups of COVID-19 outpatients **(A)**; the late-female (12 samples from 10 donors) and late-male (29 samples from 21 donors) sub-groups of COVID-19 outpatients **(B)**. The bar graphs show the contribution of variables for both dimensions (1 and 2) in the PCA analyses. **(C, D)** Correlation heatmap shows the Spearman’s r values between the complete set of variables analyzed for early-female (20 samples from 19 donors) and early-male severe (22 samples from 21 donors) sub-groups of outpatients **(C)**; or for late-female (12 samples from 10 donors) and late-male (29 samples from 21 donors) sub-groups of outpatients **(D)**. A scale of blue-to-red color indicates a negative-to-positive correlation. **(E, F)** Circos plot shows significant Spearman correlations without adjustment between the pairs of variables displayed in **(C)** or **(D)** for early-female and early-male sub-groups of outpatients **(E)**; or for late-female and late-male sub-groups of outpatients **(F)**. The color of the links represents the magnitude of Spearman’s r values. **(G, H)** Polar plots showing the differential composition of humoral immune responses at different sub-groups. Each bar represents the mean z-scores of variables for early-female (20 samples from 19 donors) or early-male outpatients (22 samples from 21 donors) **(G)**; and late-female (12 samples from 10 donors) or late-male outpatients (29 samples from 21 donors) **(H)**.

Heatmaps in **Figures 2**–**5** were generated using the package GGally (v 2.1.1) in R studio to represent the correlation between all variables analyzed.

### Correlation Analyses

We calculated the Spearman correlation and their p values using the R package “correlation” (v 0.6.0) in R Studio. For **Figures 2** and **3** this analysis was performed using Holm adjust with c.i. 0.95. In **Figures 4**, **5** this analysis was performed without adjusting. The p values <0.05 were highlighted on the heatmap with an asterisk and subsequently used to create a circos plot with the R package “circlize” (v 0.4.12) in R Studio.

### Principal Component Analyses

Unsupervised principal components analyses (PCA) were performed in R Studio. Complete data were scaled to variance units using FactoMineR (v2.4 R studio) and the PCA results were extracted and visualized using factoextra (v1.0.7 R Studio). For **Figures 2**, **3**, the PCA was performed using all samples data and graphed by days from the onset of symptoms (Early ≤ 43 days; Late > 43 days) for **Figure 2** or by the severity of symptoms (mild or more severe) for **Figure 3**. In **Figures 4**, **5** the data was divided into two subgroups by the days from the onset of symptoms criteria and then the PCA was performed using severity of symptoms for **Figure 4** or sex for **Figure 5**.

### Polar Plots

Polar plots represent the value of different variables normalized to the Z-score of data. Then, to create the different polar plots in **Figures 2**–**5** the z-score mean of variables was calculated using considering the samples from each subgroup.

### Study Approval

Human blood samples were collected after signed informed consent that was obtained in accordance with protocols and approval from the Institutional Review Board (Valdivia Health Service). Written informed consent was received prior to participation.

## Results

### Cohort Description

A total of 83 blood samples were collected from 70 COVID-19 outpatients between 4 and 201 days following symptoms onset, in addition, seven subjects provided longitudinal blood samples.

Specifically, from 70 subjects, 67 tested positive for SARS-CoV-2 by nasopharyngeal PCR, two tested positive by serological IgM/IgM-IgG test and one was diagnosed based on clinical manifestations of COVID-19. All of these patients recovered without the need for hospitalization. These individuals were recruited during the first wave of the COVID-19 pandemic between July and December 2020 in southern Chile. The cohort was composed by 29 female and 41 male donors (41.4 and 58.6% respectively), the complete set of 83 donations was composed of 32 samples from women and 51 from men (38.6 and 61.4% respectively). While the mean age of donors across 83 samples was 34, mean age of females and males at their sampling was 32 and 36 years old respectively (**Table 1**).

**Table 1:** Demographics and clinical characteristics of the cohort.

		First donation (n=70)	All samples (n=83)
Sex (n, %)	Female	29 (41.4%)	32 (38.6%)
	Male	41 (58.6%)	51 (61.4%)
			
Age (Years, mean ± SD)	All	34.26 ± 9.51	34.61 ± 9.68
	Female	32.34 ± 9.94	31.75 ± 9.69
	Male	35.61 ± 9.08	36.41 ± 9.33
			
Days from symptom onset to enrollment (mean, range)		48.99 (4-201)	58.47 (4-201)
Symptoms (n, %)	Fever	28 (40%)	
	Chills	9 (12.9%)	
	Headache	48 (68.6%)	
	Anosmia	46 (65.7%)	
	Dysgeusia	39 (55.7%)	
	Cough	33 (47.1%)	
	Odynophagia	24 (34.3%)	
	Dyspnoea	16 (22.9%)	
	Myalgia	37 (52.9%)	
	Diarrhea	19 (27.1%)	
	None	2 (2.9%)	

From the total of 83 samples, 36 were obtained from a common outbreak affecting health workers from the Hospital of Osorno City in Los Lagos Region and 47 samples were obtained from convalescent blood donors at Hospital Base of Valdivia city in Los Ríos region (43.4 and 56.6% respectively).

As detailed below, a broad range of symptoms was reported (**Table 1**). A survey was carried out at the time of recruitment to assign a clinical severity score according to the symptoms when acute disease was reported.

### Convalescent COVID-19 Patients Exhibit Diverse Humoral Immune Profiles

To broadly explore the humoral immune responses mounted by COVID-19 outpatients against SARS-CoV-2 spike protein, we profiled serum samples obtained from convalescent individuals described above. We assessed 14 serological and cellular features including Ab-dependent NK cell activation. Due to the polyclonal nature of a humoral response, multiple features may simultaneously contribute to a differential control and clearance of infection. Thus, we determined circulating titers and functional features of SARS-CoV-2 specific Abs that recognize spike protein and the spike-derived receptor binding domain (RBD). Levels of Ab isotypes and IgG subclasses together with their neutralizing function, as well as NK cell activation and degranulation functions were measured. Heterogeneous responses were observed across individuals, as depicted by Z-scores (**Figure 1A**).

Specifically, we observed that all convalescent patients had seroconverted, presenting positive spike-specific IgG (spike-IgG) titers with area under curve (AUC) values higher than the mean response of healthy donors plus two standard deviations (**Supplementary Figures 1A, B**). A predominant anti-spike IgG response was observed across convalescent donors in comparison to spike-IgM or IgA titers. In general, RBD-specific IgG, IgM, and IgA titers were lower than the corresponding spike-specific titers (**Figure 1B** and **Supplementary Figures 1A, B**).

Interestingly, filtering statistically significant p values with Holm-Bonferroni algorithm, a stronger correlation between RBD- and spike-specific Ig titers were found with IgA (r=0.86, p<0.00001), as compared to IgM (r=0.8, p<0.00001) or IgG (r=0.75, p<0.00001) Ab isotypes (**Supplementary Figure 1C**). This suggests that circulating IgA Abs against full spike protein, are mainly directed to the RBD region. In addition, high titers of IgG, IgA, and IgM against the spike protein were observed in samples obtained during the first six weeks since symptoms onset. However, after six weeks we observed a stabilization of IgG and IgA titers (plateau curves), along with a rapid waning of IgM titers (**Figure 1B**).

Due to the potential for different IgG subclasses to differentially activate Fc-gamma receptor (FcgR) mediated functions, we profiled IgG subclasses. IgG1 and IgG3 were the predominant spike-specific IgG sub-classes, while IgG2 and IgG4 were less abundant in the cohort (**Figure 1C** and **Supplementary Figures 1D, E**). These findings are in line with previous reports (55–57), and were also supported by the results of our longitudinal samples (**Supplementary Figure 1D**). Although detection of IgG1 and IgG3 presented similar kinetics over time since the onset of symptoms, 80 out of 83 samples of the cohort had detectable titers for spike-specific IgG1 (96.4%) and 75 samples presented seroconversion for spike-specific IgG3 (90.4%). Spike-specific IgG2 and IgG4 titers slightly rose across the samples obtained during the first six weeks (39.8% and 67.5% respectively). These results suggest an active IgG class-switching in COVID-19 convalescent outpatients mainly towards IgG1.

Similar results were observed in the analysis of Ab functionality, where it was possible to measure Ab neutralizing titers and NK cell effector functions in most analyzed subjects (**Figure 1D**). Specifically, we assessed three different Ab-dependent NK cell-effector functions that are related to their activation: CD107a upregulation and macrophage inflammatory protein 1β (MIP1β) and interferon γ (IFNγ) secretion (58). We observed that the majority of convalescent samples induced MIP1β^+^ and CD107a^+^ NK cell activation, despite some samples being collected up to 6 months from symptoms onset (88% and 95.2% respectively). The same trend was found for IFNγ secretion, where most of the samples displayed positive activity (78.3%) (**Figure 1D**). Similarly, neutralizing Ab titers expressed as NT50, the reciprocal value of the respective serum dilution able to inhibit 50% of infection, displayed a broad range of neutralization activities across the cohort (mean NT50 = 1454.9), with samples presenting null NT50 values (2 out of 83, 2.41%), some over 1000 (34 out of 83, 40.96%) and others over 5000 (5 out of 83, 6.02%) (**Figure 1D** and **Supplementary Figure 1F**). Altogether, these data show that neutralization activity and Ab-dependent NK cell functions are key features of the anti-SARS-CoV-2 spike-specific humoral response developed in non-hospitalized COVID-19 convalescents, which are sustained up to six months during the convalescence period in COVID-19 outpatients.

### Diverse Igs Correlate With Functional Features Mounted Against SARS-CoV-2 Spike Protein

To assess the potential contribution of RBD and spike-specific IgA, IgM, and IgG titers to Ab functionality from convalescents sera, neutralizing and NK-dependent Ab functions from each patient were compared to Ab subclass and subtype abundance in each patient across the cohort (**Figure 2A**). Overall, we found stronger correlations between spike- and RBD-specific IgM and IgA titers with neutralizing activity compared to NK-effector functions. A correlation chart (**Figure 2B**) illustrates the relationship between Ab titers and the functional activities mounted against spike protein. We observed strong associations between spike-specific IgM and IgA titers (r=0.75 p<0.00001), between RBD-specific IgM and IgA (r=0.68 p<0.00001) and also between spike-specific IgG1 and IgG3 (r=0.71 p<0.00001). Each correlation with a p value lower than 0.05 is displayed in a circos plot analysis (**Figure 2C**).

NK cell activation markers CD107a upregulation, MIP1β, and IFNγ secretion correlated with RBD-specific IgM, RBD-specific IgA titers, and spike-specific IgM titers (**Figures 2A, B**).

In addition, because spike-specific IgG titers were high, even in those samples that were taken up to 6 months from symptoms onset, their contribution to NK function should be considered regardless of the low correlation value observed (**Figures 2A, B**), especially when we consider that non-neutralizing function relies on the avidity of the Ab response (59).

On the other hand, every spike-specific Igs subtype correlated with NT50, as seen in **Figures 2A, B**, suggesting that the broad composition of the humoral responses contributes at least partially to this function. Correlations between NT50 and spike-specific IgG, IgM or IgA titers were found to be stronger than those between NT50 and RBD-specific Ig titers (**Figures 2A, B**). This observation suggests that IgG may mediate neutralization, but not only by targeting epitopes within the RBD region.

A strong correlation between IgM titers and NT50 values (r=0.72 p<0.00001 for spike-IgM and r=0.62 p<0.00001 for RBD-IgM titers) (**Figures 2A, B**) highlights their critical contribution to SARS-CoV-2 neutralization, especially during the first six weeks from symptoms onset. Likewise, circulating spike- and RBD-IgA titers strongly correlated with NT50 values (r=0.65 p<0.00001 and r=0.6 p<0.00001, respectively) (**Figures 2A, B**), suggesting that RBD-specific IgM and IgA titers significantly contribute to SARS-CoV-2 neutralization, at least during the early convalescence of COVID-19 outpatients. In addition, the high magnitude of IgG titers, together with their higher seroprevalence and the correlation between spike-IgG and NT50 (r=0.45 p<0.01) (**Figures 2A, B**) support its contribution to neutralization, as compared to RBD-IgG titers. Furthermore, IgG subclass titers were also compared to NT50 values, and only IgG1 and IgG3 positively correlated with neutralization (**Figure 2B**). Overall, these results point to a functional and dynamic humoral response directed against spike protein in outpatients who overcame COVID-19.

### Time-Dependent Ab Functional Profiles

To further explore the dynamic nature of the humoral response throughout convalescence in outpatients, we stratified individuals according to the time of sampling (Early ≤ 43 days; Late > 43 days since the onset of symptoms) and performed a principal component analysis (PCA) including each variable assayed (**Figure 2D**). The results of this analysis show that both Dimensions (Dim1 and Dim2) capture 49.2% of the variance of variables, and features such as Ab-dependent NK cells functions, neutralization, spike-IgM, and RBD-IgM contribute the most to the stratification of individuals (**Figure 2E**). To further explore this concept, we generated a correlation chart (**Figure 2F**) and conducted a circos plot analyses (**Figure 2G**), plotting each variable by using polar plot analysis for these two sub-groups (**Figure 2H**). Multiple and strong correlations of neutralizing activity with spike- and RBD-IgM, spike-IgG, and RBD-IgA present in early samples were not found in late samples, suggesting that those remaining Ig titers present during late convalescence do not sustain long term neutralization. Polar plots confirmed the strong contribution of neutralizing and NK cell activities in samples obtained during the first 43 days after symptoms onset, and also, a profile of a long-term sustained NK cells activity, with less neutralization activity in late convalescence samples. These polar plots also confirmed the preferential long-term prevalence of spike and RBD-specific IgG titers.

Taken together, these results suggest that spike-specific Ab-dependent NK degranulation and activation functions evolve early in convalescent COVID-19 patients, potentially synergizing with the highly heterogeneous and neutralizing activities present during the months following infection. As neutralizing titers are known to be enriched in patients with severe COVID-19, it is also highly likely that Ab-dependent NK effector functions play a critical role in the mechanisms underlying the recovery of COVID-19 patients and long-term functions of the humoral response mounted against SARS-CoV-2.

### Ab Mediated NK Function Correlates With Mild Disease and May Influence Morbidity in COVID-19 Outpatients

Next, we wanted to investigate whether characteristics of the humoral response could be linked to symptoms presentation and disease morbidity. To this end, we evaluated the clinical records of patients that were registered since recruitment, which included 10 different symptoms and a severity score compiled during acute disease. While no single symptom was associated with higher neutralizing or Ab-dependent NK effector functions (**Figure 3A** and **Supplementary Figure 2**), some symptoms were observed more frequently in the cohort (**Table 1**). Next, we classified patients according to severity score which included the number and magnitude of symptoms. Severity scores ranked between 0 to 2 for mild disease and between 3 to 5 for more severe conditions. Although convalescent patients with lower and higher severity scores were indistinguishable from each other in terms of neutralizing, spike- or RBD-specific IgG titers (**Figure 3B**), differences between patient groups were evident when analyzing their NK effector functions (**Figure 3B**). This observation was further validated by a principal component analysis (PCA), where NK functions segregated mild from more severe COVID-19 outpatients (**Figures 3C, D**).

The correlation chart and the circos plot analysis showed that the higher NK activity displayed in mild patients was correlated with spike-IgM and also CD107a was correlated with spike-IgG. In contrast in more severe outpatients, NK activity was not correlated with any Ig titer (**Figures 3E, F**). In addition, polar plot analysis revealed that in patients with milder conditions, NK activity was overrepresented as compared to neutralization, however, the opposite was observed in more severe outpatients (**Figure 3G**).

To expand the analysis, we included the studied variables in new PCAs, applied separately to samples obtained during early or late convalescence (**Figures 4A, B**). These analyses allowed for the examination of convalescent patients who suffered mild or more severe symptoms due to a differential contribution of Ab-dependent NK functions during early (**Figure 4A**) and late convalescent periods (**Figure 4B**). Correlation analysis (**Figures 4C–F**) showed that in contrast to early-mild subjects, early-more severe patients have no significant correlations between spike-IgM, spike-IgG, or RBD-IgG and Ab-dependent NK effector functions, (**Figures 4E, F**). These analyses also show that late-more severe patients have no significant correlations between RBD-specific IgA and any Ab function measured (**Figures 4D, F**).

Polar plot analyses (**Figures 4G, H**) showed the profiles of mean values of the data analyzed and highlights the differences observed in Ab-mediated NK cell function between mild and more severe patients in early and late convalescence. Interestingly, higher Ab-dependent NK effector functions were found in patients with mild symptoms regardless of early and late convalescence. Moreover, the most prominent difference between mild and more severe subjects in early convalescence was the higher levels of Fc-mediated NK activity in mild individuals (**Figure 4G**). In comparing more severe individuals at early and late convalescence, we observed relatively diminished levels of neutralization activity, along with decreasing levels of IgA and IgM targeting spike and RBD, during late convalescence. Interestingly, spike and RBD IgG levels were maintained during early and late convalescence (**Figures 4G, H**). These findings suggest that viral neutralization at early convalescence in more severe samples was primarily mediated by IgA and IgM.

In order to further dissect the mild-disease associated characteristics of convalescent sera, we compared Ab features based on their demographics. Sex is known as an important variable in COVID-19 (60, 61), as men present more severe symptoms. Through principal component analyses, we observed a trend towards the differential contribution of Ab-dependent NK effector functions in female and male samples obtained during early convalescence, while less evident differential contributions were observed in late convalescence samples (**Figures 5A, B**).

To better examine the differential contribution of the studied Ab titers and functions in men and women, multiple correlations between variables were evaluated after segregating samples as before, in the early and late convalescent periods (**Figures 5C, D**). In general, as drawn in circos plots, the evaluated Igs correlated less with the Ab-dependent NK functions in women than in men (**Figures 5E, F**). While polar plot analyses revealed that, besides presenting a profile of higher neutralizing ability, men also presented higher spike and RBD-specific IgM and IgG titers compared to women in early samples (**Figure 5G**). However, a late convalescence profile similar to late-more severe samples was observed in males (**Figure 5H**). This common profile was drawn by lower NK-effector functions and higher spike and RBD-specific IgG titers than those found in late convalescent female samples. These results suggest that the abundance of those spike- or RBD-IgG titers do not reflect their Fc Receptor functionality, suggesting that less prevalent Ab-dependent NK effector functions may influence COVID-19 morbidity.

## Discussion

As COVID-19 pandemic continues, more information regarding the human immune response against SARS-CoV-2 has been unveiled. The study of the responses mounted by convalescent individuals against the virus is critical to better characterize effective naturally evolved strategies to overcome disease. The relevance of this new information is highlighted if the limited scope of prophylactic vaccination efforts in developing countries is taken in count (62). For instance, regarding the extent of long-term responses, even without segregation of individuals due to the severity of their COVID-19 symptoms, it has been shown that the majority of SARS-CoV-2 infected individuals develop serum Abs that last for several months (63–65), even beyond a year after infection (66, 67). Our results regarding outpatients support those previous findings and are consistent with the waning of IgM and stabilization of IgA and IgG titers of still positive neutralizing titers found after several months post-symptoms onset (64, 68–70). Interestingly, Marcotte H et al. have found a similar seroprevalence of IgG titers over time, even up to 15 months after infection (67), which are in the same trend of results obtained by our study.

A typical antiviral response induces neutralizing Abs that can inhibit infection. This humoral function has been extensively evaluated as a correlate for protection against SARS-CoV-2 infection (32–34, 64, 71). However, once the virus has become widespread in the host, additional Ab functions may be required for efficient viral clearance. To date, Fc-effector functions have not been extensively evaluated in the humoral response mounted against SARS-CoV-2. Consistent with the potential Fc-dependent effector functions contributing to SARS CoV-2 humoral responses, we have found persisting spike-specific Ab-dependent NK effector functions detectable up to six months from the onset of symptoms in COVID-19 outpatients. In line with these findings, several studies have shown an enrichment of Fc receptor binding and Fc-effector functions in acute COVID-19 (18, 27, 72). Furthermore, Zohar et al. described an enrichment of Fc-effector functions in COVID-19 survivors as compared to deceased individuals (19). These studies highlight the relevance of Ab-dependent effector functions in viral clearance and pose the question of how these functions arise during acute SARS-CoV-2 infection.

Although this is not a longitudinal study, our findings support an important role for Fc-mediated NK activities in controlling SARS-CoV-2 infection. We show that Ab-dependent NK-effector functions were enriched in samples from outpatients who suffered milder symptoms compared to those presenting with more severe disease, with Ab-dependent NK functions more highly sustained for at least six months after acute infection, as compared to neutralizing activity over the same period. In line with our results, Lee WS et al. showed that Ab-dependent cellular cytotoxicity (ADCC) and Ab-dependent phagocytosis (ADCP) Fc-effector functions were preserved up to five months from symptoms onset (73). Also, supporting the relevance of NK cell effector functions at effective immune responses against this infection, Notarbartolo S et al. showed that NK cell population is significantly expanded in individuals with mild disease (17), plausibly synergizing with the Fc-dependent characteristics of Abs. Additionally, NK cell-based therapy strategies, have now received attention to hopefully control the harmful cytokine storm induced by SARS-CoV-2 activation of infected macrophages and CD4+ T cells in severe COVID-19 patients (74–77).

The role of Abs in protection against reinfection is another scenario. Previous studies had determined the association between Ab development and a decreased risk of reinfection with SARS-CoV-2, but the information is still limited (65, 78, 79). Furthermore, the low rate of reinfection reported so far across COVID-19 convalescent individuals (80, 81), suggests that other Ab functions, beyond neutralization, such as sustained Ab-dependent NK effector functions could contribute to long-term protection. In support of this, Fc-effector functions have been correlated with prophylactic protection against COVID-19 (47, 48) and are required for optimal protection during post-exposure therapy conferred by neutralizing human monoclonal Abs and convalescent plasma against SARS-CoV-2 (82, 83). Taken together, these results suggest that Fc-effector functions not only contribute to mounting effective immune responses against acute SARS-CoV-2 infection but may also contribute to protection against reinfection. Interestingly, a recent comparative evolutionary analysis of SARS-CoV-2 and close relatives suggests that reinfection with SARS-CoV-2 is likely to occur after a median of 16 months (84). Thus there is an urgent need to focus research beyond neutralization to further help our temporally limited understanding of the underlying mechanisms of sustained protection.

Despite the relatively small cohort size, different profiles in sera composition and function were found between subtly different sub-groups of individuals, thus demonstrating the highly diverse range of responses elicited against SARS-CoV-2 infection, even in outpatients. Although clear differences in functional features were observed between mild and more severe cases, the similar Ig profile suggests that a differential functionality in titers, rather than their abundance, is critical for responses in individuals with phenotypically mild COVID-19. In support of this, a similar profile of reduced: specific NK-effector functions, neutralization, and IgM & IgA titers were found in male individuals with more severe symptoms during late convalescence. Although further studies are needed to substantiate this, the data suggest that in spike- and RBD-specific IgG responses, disrupted polyfunctionality of spike-specific Ig titers is detrimental for COVID-19 resolution, which is strongly supported by a female sex-dependent enrichment of Fc effector functions.

Strict Holm-Bonferroni-filtered correlations were found between Ab-dependent NK effector and neutralization functions preferentially in mild cases thus supporting the relevance of a broad and polyfunctional response in mild COVID-19 cases. Similar data were obtained for correlations between those Fc-effector functions and RBD-IgM, spike-IgM, or spike-IgG titers.

Notably, regarding the persistent occurrence of SARS-CoV-2 variants of concern (VOC), recent work from Bartsch Y et al. shows that while a loss of neutralizing activity against Omicron VOC occurs in vaccinated individuals, it is accompanied by persisting Spike-specific antibody binding to Fc-receptors (85), further supporting the relevance of studying the contribution of non-neutralizing Ab function to protection across vaccinated and non-vaccinated individuals.

In summary, our data suggest that a polyfunctional response against spike which involves Ab-dependent-NK activity is best associated with a mild symptoms course and associated with disease resolution. These data also support the concept that outpatients, the most representative COVID-19 convalescent population, develop rapid and long-lasting spike-specific Ab-dependent NK effector functions. Whether this NK function signature contributes to immunity against future reinfection remains to be elucidated; however, if it does, it would represent a complement for neutralizing activity reported for COVID-19 convalescents and serve also as a good immune correlate to study protection after infection and prophylactic approaches.

## Data Availability Statement

The original contributions presented in the study are included in the article/**Supplementary Material**, further inquiries can be directed to Maria Ines Barria, maria.barriac@uss.cl.

## Ethics Statement

The studies involving human participants were reviewed and approved by Valdivia Health Service. The patients/participants provided their written informed consent to participate in this study

## Author Contributions

MB, MC, RA, and JG contributed to the study design and data interpretation. MC, VT, YP, MR, and COVID-19 South Chile Group contributed to clinical management, patient recruitment and data collection. MB, AG, AO-A, FB, NR, NZ, JG, and FF-V contributed to sample processing, and performing experiments. MM, JG, and FF-V contributed to statistical analysis and data visualization. MB, FK, RA, RS-R, FV-E, FB, MM, JG, and FF-V contributed to revision of the manuscript. MB, FK, FA, and JG contributed to assay development. All of the authors met authorship criteria and approved the publication.

## Funding

This work was supported by National Agency of Research and Development (ANID) projects COVID0422 (to MB), FONDEF ID20I10192 and ID18I10261 (to MB). FONDECYT 1190156 (to RS-R), 1180798 (to FV-E) and 3200913 (to FF-V). Proyecto FIC20-10 Gobierno Regional de Los Ríos (to VT). We also thank FONDEQUIP EQM150061. Reagents in the Krammer laboratory were generated with support of the NIAID Collaborative Influenza Vaccine Innovation Centers (CIVIC) contract 75N93019C00051, NIAID Center of Excellence for Influenza Research and Surveillance (CEIRS, contract # HHSN272201400008C), generous support of the JPB Foundation and the Open Philanthropy Project (research grant 2020-215611) (5384); and by anonymous donors.

## Conflict of Interest

Authors JLG and RA were employed by company Ichor Biologics LLC. JLG and RA were partially supported by Ichor Biologics LLC.

The remaining authors declare that the research was conducted in the absence of any commercial or financial relationships that could be construed as a potential conflict of interest.

## Publisher’s Note

All claims expressed in this article are solely those of the authors and do not necessarily represent those of their affiliated organizations, or those of the publisher, the editors and the reviewers. Any product that may be evaluated in this article, or claim that may be made by its manufacturer, is not guaranteed or endorsed by the publisher.
