# Supplementary material for: Sustained Antibody-Dependent NK Cell Functions in Mild COVID-19 Outpatients During Convalescence
Source: Front Immunol. 2022 Feb 7;13:796481. doi: 10.3389/fimmu.2022.796481 (PMC8859986; doi:10.3389/fimmu.2022.796481)
Supplement: Supplementary file 1 [file DataSheet_1.pdf]

Supplementary Material

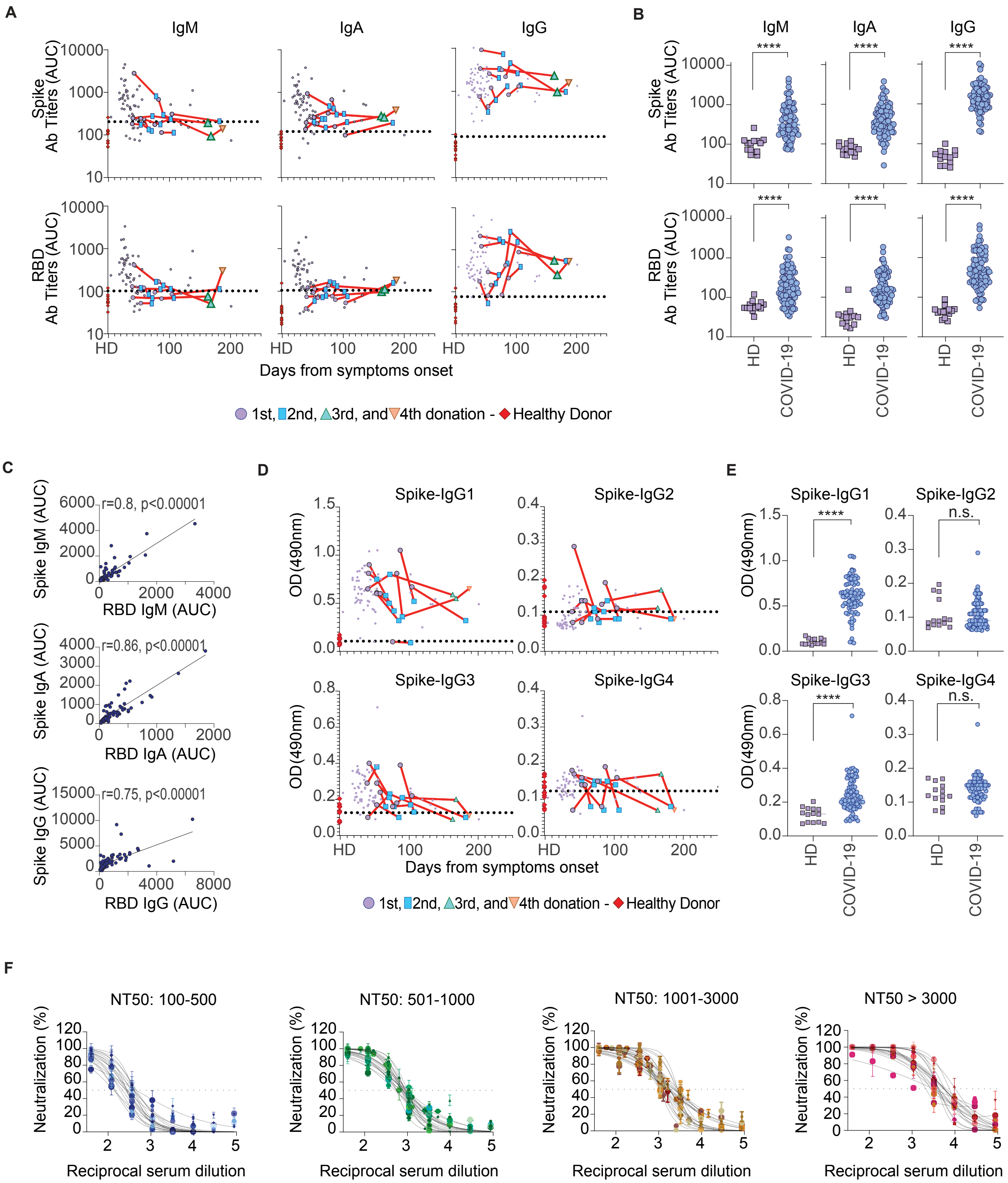

Supplementary Figure 1. Humoral response mounted against SARS-CoV-2 Spike is represented up to 6 months from the onset of symptoms in COVID-19 outpatients. (**A**) Spike and RBD-specific titers for IgM, IgA and IgG antibodies measured as AUC (Area Under Curve) are displayed according to their respective sampling day from the onset of symptoms (83 COVID-19 samples from 70 donors and 14 controls). 13 follow-up samples are distinguished with different shapes and linked with a red trace. Healthy donor threshold is shown (Mean plus 2 standard deviations). (**B**) Spike and RBD-specific titers for IgM, IgA and IgG antibodies measured as AUC are compared between convalescent outpatients and healthy donors. Two-tailed unpaired nonparametric Mann-Whitney test $p<0.0001$ are shown as ****. (**C**) Correlations between RBD- and spike-specific titers for IgM, IgA and IgG subtypes for the entire cohort of samples (83 samples from 70 donors). Spearman's r and p values are shown. (**D**) Spike-specific IgG1, IgG2, IgG3 and IgG4 sub-class Abs measured as OD490 values are displayed according to their respective sampling day from the onset of symptoms. 13 follow-up samples are distinguished with different shapes and linked with a red trace. Mean values of healthy donors are shown as dotted lines (83 COVID-19 samples from 70 donors and the mean of controls). (**E**) Spike-specific IgG1, IgG2, IgG3 and IgG4 sub-class Abs measured as OD490 values are compared between convalescent outpatients and healthy donors. Two-tailed unpaired nonparametric Mann-Whitney test $p<0.0001$ are shown as ****. (**F**) Neutralization curves using HIV-1-S Δ19 pseudovirus. Samples were titrated in triplicate at serial threefold dilutions (1:40 to 1:87,480) and are expressed as percent of neutralization ± %CV. It displays the NT50 range above each graph.

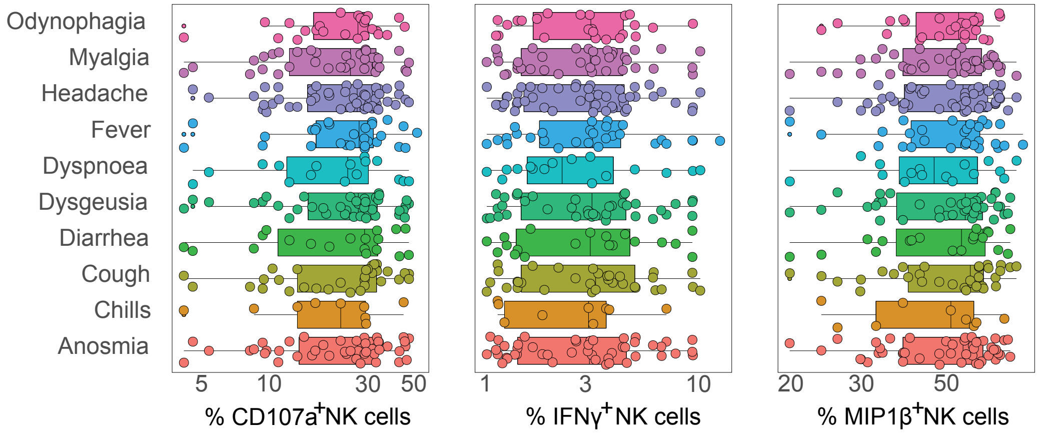

Supplementary Figure 2. Ab-dependent NK effector functions and symptoms reported at the time of diagnosis. The box plots show the Ab-dependent NK effector function activity grouped according to different symptoms reported by patients at the time of diagnosis (70 donors).
